# Development of an MFL Coil Sensor for Testing Pipes in Extreme Temperature Conditions

**DOI:** 10.3390/s21093033

**Published:** 2021-04-26

**Authors:** Nagu Sathappan, Mohammad Osman Tokhi, Liam Penaluna, Zhangfang Zhao, Fang Duan, Gholamhossein Shirkoohi, Aman Kaur

**Affiliations:** 1 School of Engineering, London South Bank University, London SE1 0AA, UK; tokhim@lsbu.ac.uk (M.O.T.); zhangfang.zhao@lsbu.ac.uk (Z.Z.); fang.duan@lsbu.ac.uk (F.D.); maziar.shirkoohi@lsbu.ac.uk (G.S.); 2 NDT Section (NSIRC), TWI, Cambridge CB21 6AL, UK; liam.penaluna@twi.co.uk; 3 London South Bank Innovation Center, Cambridge CB21 6AL, UK; kaura13@lsbu.ac.uk

**Keywords:** coil sensor, magnetic flux leakage testing, magnetic field, corrosion monitoring

## Abstract

This paper aims to design a coil sensor for corrosion monitoring of industrial pipes that could detect variations in thickness using the MFL (Magnetic Flux Leakage) technique. An MFL coil sensor is designed and tested with pipe sample thicknesses of 2, 4, 6, and 8 mm based on the magnetic field effect of ferrite cores. Moreover, a measurement setup for analysing pipe samples up to a temperature of 200° Celsius is suggested. Experimental results reveal that the MFL coil sensor can fulfil the requirements for MFL testing of pipes in high temperature conditions, and that the precision of MFL monitoring of pipes to detect corrosion at high temperatures can be improved significantly.

## 1. Introduction

Pipelines constitute an important mode of transportation for oil and gas, where bound parts and components must work in hostile environments in industries such as nuclear, solar thermal, and oil. Pipes, tanks, pressure vessels, and absorbent tubes containing flammable liquids will develop defects such as creep, thermomechanical fatigue, and hot corrosion as a result of high temperatures [1,2,3,4,5,6]. Local pitting corrosion can initiate stress corrosion cracking or result in small-scale leaks, which is another common problem that industrial pipes can face [7,8,9]. The absorber tubes’ harsh operating environment, which includes high temperatures (400–550 °C), contraction/expansion, and vibration, can cause creep, thermo-mechanical fatigue, and hot corrosion [10]. This may cause the internal or external structure of the parts and components to collapse, resulting in the plant’s closure, economic damage, and in some cases, a serious threat to human life [1]. Pitting corrosion and stress corrosion cracking are also common problems with stainless steel pipes [9]. Thus, condition monitoring of industrial pipes is of crucial importance and is a very challenging task. The use of Non-Destructive Testing (NDT) techniques, can reduce the likelihood of the structures being drained [1]. The operating conditions with regard to the location, access, size of the system, and structural complexity of the part under monitoring are all factors that restrict the extent to which NDT techniques can be used. Various NDT techniques, including Acoustic Emission (AE), Eddy Current (EC), Thermography, optical techniques, and laser and conventional Ultrasonic Testing (UT) have been applied at up to 300 °C with qualitative results and sensitivity to noise in laboratory settings [6]. Guided Wave Testing (GWT) is a modern NDT technique that has been widely used to assess the structural integrity of large structures in oil/gas, nuclear, and solar thermal industries [10]. Conventional UT and GWT can also be used to inspect high-temperature structures, but the reliability of these methods is restricted by the transducers’ high-temperature output [10,11]. AE has been commonly used in bridges, nuclear plants, turbines, and aerospace metallic systems for structural health monitoring (SHM) [12,13,14,15,16,17,18,19]. Piezoelectric transducers such as Yttrium Calcium Oxyborate and Aluminum Nitride have been used with AE at up to 700 °C and 1200 °C, respectively [20,21]. Background noise is a problem in AE analysis, and it has an effect on the output quality. Eddy Current Testing (ECT) is a non-contact technique in which a dynamic magnetic field is produced around the coil, causing eddy current on the surface of the specimen that is at proximity to the coil [22,23]. It has also been used at high temperatures up to 500 °C. Industrial high-temperature EC probes are available that can run continuously at temperatures between 280 and 380 °C [24,25]. However, EC testing consumes a large amount of time in scanning large pipe structures. Urayama et al. [25] have developed and tested a dual Electro-Magnetic Acoustic Transducer (EMAT)/EC probe for monitoring pipe wall thinning at high temperatures (300 °C). It is a non-contact defect detection technique [26] that uses laser-generated ultrasound [27] where an array of EMATs is used for crack detection using lamb waves, and a qualitative model is developed for the laser-generated transient ultrasonic lamb waves in orthographic thin plates [28,29]. This technique, however, cannot be used to detect corrosion.

Magnetic Flux Leakage (MFL) is one of the most widely used NDT technique that can detect a defect in both the axial and circumferential directions, though it is susceptible to pipe wall thickness and other factors such as defect form [30,31]. Magnetic sensors have advanced significantly with the advancement of the semiconductor electronics industry, removing the limitation of measuring instruments to magnetic particle inspection [30,31,32]. Shen et al. [30] have designed an Electro-Magnetic (EM) sensor to non-destructively monitor phase (microstructure) transformation in strip steels during the cooling process following hot rolling [30]. The process necessitates a precise analysis of EM sensor signals as well as the ability to predict signals from desired microstructures at the appropriate temperatures. The system, however, is not suitable for corrosion monitoring. Thus, the design of an MFL sensor for high temperature testing necessitates a detailed study of EM sensor signals as well as the ability to predict signals from desired microstructures at specific temperatures [30]. Unlike other induction coil sensors that are found to be effective at room temperature testing, it can guide more magnetic flux to leak out. The MFL coil sensor is found to be suitable for corrosion monitoring at defined temperature conditions.

## 2. Principle of MFL Testing

**Figure 1:**
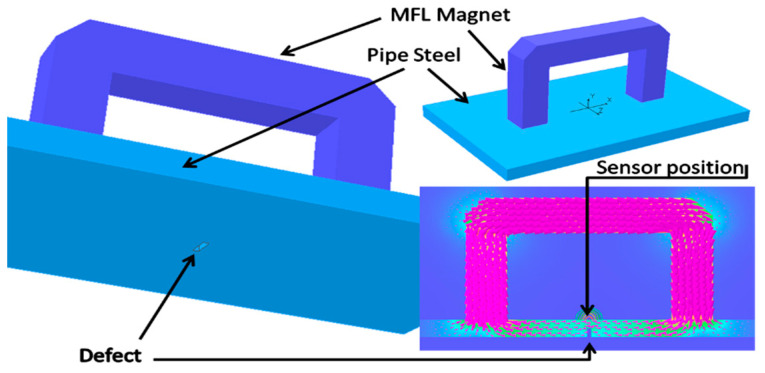
Basic principle of MFL inspection.

A permanent magnet equipped with a magnetic sensor placed in the centre of the pipe sample will create a magnetic field around the circumference of the pipe wall, with magnetic lines of flux in the axial direction as depicted in Figure 1. If there is no corrosion on the wall, the magnetic lines of flux will move through the wall inside. However, if there is corrosion on the wall, the magnetic lines of flux will deform and the remaining flux will spread out of the pipe wall and cause the leakage of flux [32].

According to Faraday’s law [33], the integral of the electric field over a closed loop is equal to the induced voltage inside the coil;

(1) $$\oint E\cdot ds=–N\frac{d\phi \left(t\right)}{dt}$$

(2) $$V_{c}\;=-N\frac{dB}{dt}$$

where *N* denotes the number of wire loops, *ϕ* denotes magnetic flux, and V_c_ denotes the induced voltage inside the coil.

## 3. Permanent Magnets and Magnetic Sensors at High Temperature

Permanent magnets are known to perform better in low temperatures than in hot environments [34]. Extreme heat causes permanent magnets to lose their magnetic strength and when a permanent magnet operates under hot conditions, the magnetic particles travel quickly and become chaotic, causing misalignment and flux losses [34]. Rao [35] lists and discusses the physical, thermal, mechanical, and magnetic properties of AlNiCo permanent magnet materials. The data gathered includes a comparison of the maximum temperature of the AlNiCo grades below which no change in metallurgical structure is observed, as well as a comparison of temperature characteristics and irreversible losses of permanent magnets at room temperature after exposure to high temperatures. The author thus explains why AlNiCo has been considered a mature product in the trade for nearly sixty years [35]. The inspection of absorber tubes necessitates the use of a non-contact NDT technique that can inspect the entire length of the tube from a single stage and can be used without a couplant at high temperatures [36]. Since the EMAT is a non-contact technique that has been used in GWT and can be used to inspect structures in hostile conditions such as moving specimens and high temperatures, it is suitable for this application. As a result, GWT can be performed with non-contact transducers that can withstand high temperatures and excite/receive guided waves, especially in the T (0.1) wave mode (or SH0) [36,37,38].

Rochaz et al. [39] have proposed a Wheatstone bridge circuit with Giant Magneto Resistance (GMR) sensor, which consists of approximately 21 magnetic layers made of Nickel-iron (NiFe) separated by a nonmagnetic material Silver (Ag). Their NiFe magnetic layer thickness is 2 nm, and the Ag spacer is 1.1 nm thick. The advantage of this GMR structure design is its stability when exposed to high temperatures as compared to other GMR sensor designs that use a Cu layer spacer. Moreover, these sensors have better output linearity, low hysteresis signal (less than 1 O_e_), and the effect of magnetic resistance decreases by a factor of 1.5 to 3 for temperatures between 4 K and room temperature [39]. With increasing temperature, the amount of electrons scattering in NM, also known as nano layers, increases, allowing the number of electrons passing through the layers of GMR structures to increase, thus decreasing the efficiency of the GMR process. Because of their high sensitivity, power transducers based on Hall sensors can be used for direct power measurement. However, their insufficient sensitivity typically necessitates the use of ferromagnetic cores to concentrate the magnetic flux into the sensor area. GMR-based sensors have higher sensitivity, making them potential Hall sensor replacements in this application and at room temperature [39].

## 4. Design of Coil Sensor

Since soft ferrites have a low coercivity, they can easily adjust their magnetization and serve as magnetic field conductors. Table 1 shows some of the properties of ferrite core used to develop the coil sensor [40].

**Figure 2:**
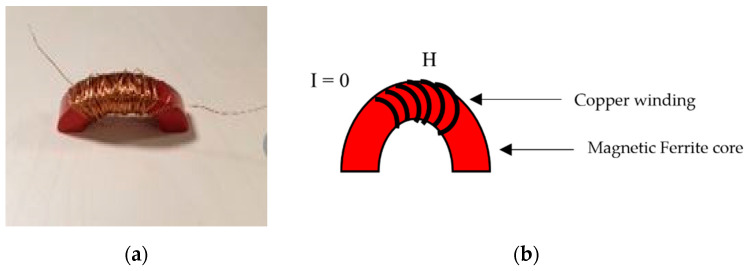
Ferrite core with the coil. (**a**) Copper coil wound around the ferrite core (**b**) Coil sensor with input current as zero.

In order to develop an open magnetic circuit, the toroidal ferrite core was cut into two halves using a grinding machine. A coil sensor, consisting of an induction coil and a ferrite core, was developed based on the magnetic field effect, as shown in Figure 2a. The ferrite core is specifically wrapped around by an enameled wire insert coil for the current to pass through it as depicted in Figure 2b. The induction coil, in contrast to the GMR sensor, should prevent saturation under a strong magnetizing field.

It is common knowledge that the MFL produced by defects can only be maximized if the ferromagnetic object is completely magnetized to saturation status. Pipe samples of various thicknesses, namely 2, 4, 6, and 8 mm were tested in this work. The material grade used was X80, and the diameter was 140 mm. MFL will be produced in the vicinity of the pipe if there are any defects, and the coil sensor will detect it. Finite element simulations of the MFL distribution affected by the ferrite core were used to investigate the magnetic field mechanism. It was noted, as shown later in this paper, that the magnetic flux is driven to leak due to the magnetic field focusing effect of the high-permeability ferrite core. With the lift-off distance of the ferrite core and the pipe sample taken into account, the magnetic flux can be directed to leak into a larger space.

## 5. Methodology and Measurement Setup for High Temperature Testing

### 5.1. Finite Element Analysis

Finite Element (FE) analysis is used for solving problems of complex geometry. A domain is subdivided (discretized) into a number of elements and analysed. The magnetic scalar potential method can be used to treat the MFL problem as a magneto-static problem. This can be expressed using the Maxwell’s equations as [41]:

(3) ∇×{H}={Js}

(4) ∇×{B}=0

where *H* denotes magnetic field power, *Js* denotes applied source current density, and *B* denotes magnetic flux density. The constitutive relation describes the behaviour of electromagnetic materials and is used to complement the field equations [41];

{B} = [µ]{H} + µ_0_{M_0_} (5)

In other region

{B} = [µ]{H}(6)

where µ_0_ is the remanent intrinsic magnetisation vector and is the magnetic permeability matrix.

**Figure 3:**
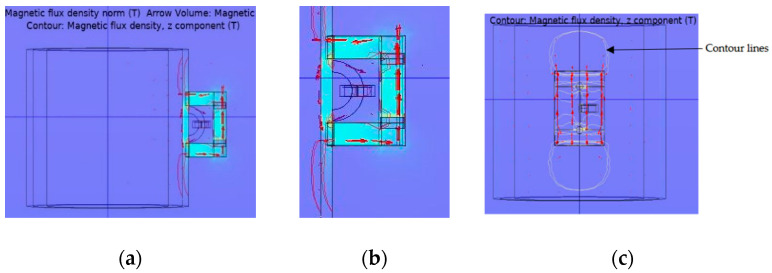
The magnetic flux pattern along the pipe in the z direction. (**a**) Magnetic flux lines from the ferrite core with the coil. (**b**) Zoom out picture of flux lines from the magnet and coil sensor. (**c**) Top view of the contour plot showing the magnetic flux flow.

A computational analysis using the FE model was conducted to design a coil sensor for detecting metal losses. Thus, the primary goal of the numerical simulation was to find the best values for the critical parameters. By using a numerical analysis instead of trial and error, not only can additional costs be avoided, but also a significant amount of time and effort can be saved. The COMSOL Multiphysics 5.4 analysis software was used for the FE analysis. For numeric simulation to consider current induction, Faraday’s law of induction, which states the relationship between the magnetic and electric fields with respect to time, is considered as a necessary condition [33]. This AC/DC module employs two physics. The magnetic flux conservation stage is used to magnetize the pipe sample with the permanent magnet. This module was used since the model was magnetized with a permanent magnet. Magnetic field with no current module was used for the coil sensor, as ferrite core with coil is used for detecting changes in pipe thickness by applying a magnetic field through the pipe. The multi-turn coil domain is used to model coils where the number of turns and coil current direction must be specified. The fine tetrahedral mesh offers a simple solution for all of the components when they are used together. Finally, since the technique is based on condition monitoring, a stationary solver was used to compute the model. The simulation can be used to calculate the magnetic flux density for each change in pipe thickness. The magnetic flux density of the Bx, By, and Bz components can be derived based on the simulation, which forms the essential operational parameters of the magnetic circuit, and the device was designed and developed. The magnetic flux flow and distribution within the pipe sample are depicted in Figure 3a. Figure 3b provides a magnified view of the magnetic circuit. The magnetic flux distribution in the model, which follows the flux flow direction, is shown in Figure 3c (magnetic flux density, z component). The contour lines in the diagram indicate the flow path of the magnetic field (Bz) portion in the z direction.

**Figure 4:**
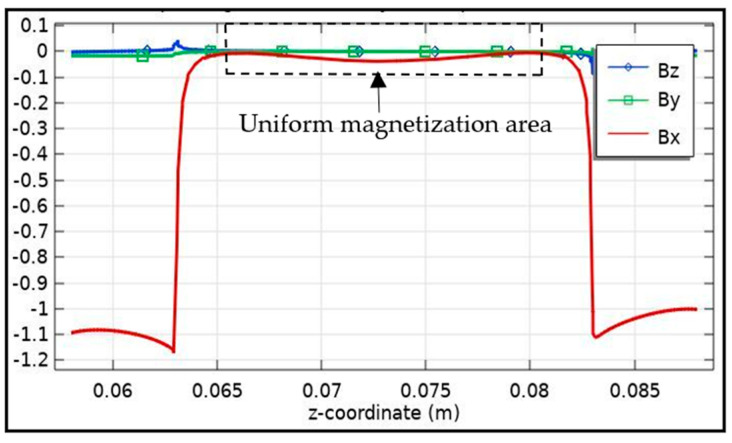
Line graph: The magnetic flux density (Bx, By, and Bz component) distribution in the pipe wall.

Figure 4 depicts the magnetic flux distribution inside the pipe wall of all the three (Bx, By, and Bz) components. The highlighted area shows the uniform magnetization area in the pipe wall. The 3D FE model is for any ferromagnetic mixed structures, as an example, the sensor response and the measured parameters can be predicted further. For each increase in pipe sample thickness, magnetic flux density values (Bz component) were obtained, from which realistic values could be predicted. Wu et al. [42] have suggested a lift off resistant MFL testing technique that includes a Helmholtz coil magnetization process as well as an entire MFL testing scheme. Additionally, to achieve 100 percent scanning coverage for drill pipes, they have suggested to divide the sensor array into two layers along with a probing device [42]. They have developed a lift-off tolerant MFL sensor consisting of an induction coil and a ferrite core and have carried out experimental tests with the sensor at various lift-off distances. They have concluded that, in contrast to traditional passive Magnetic Flux Leakage sensing, the proposed active methodology would enable the sensors to be positioned at better and greater lift-off distance in relation to the test specimen [42].

Wu et al. [42] have implemented their simulation procedures in electro-magnetic simulation software ANSOFT where the axial magnetic flux density distribution for the pipe wall is calculated. Comparable simulation results are obtained in the current work using COMSOL Multiphysics analysis software.

### 5.2. Experimental Set Up

The scale of Alnico5 grade type 54 × 83 × 70 mm [43] was chosen because it has some excellent characteristics at different temperatures. Its maximum operating temperature is 500 °C, making it simple to monitor at higher temperatures. The pull force of the AlNiCo magnet is 47 kg indicating the maximum magnetic force that the magnet will bear on a steel surface to be measured in relation to the magnetic sensor.

**Figure 5:**
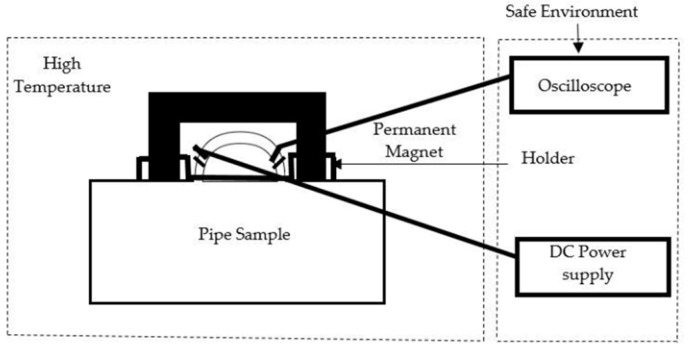
Schematic of experimental Set up for high temperature tests.

**Figure 6:**
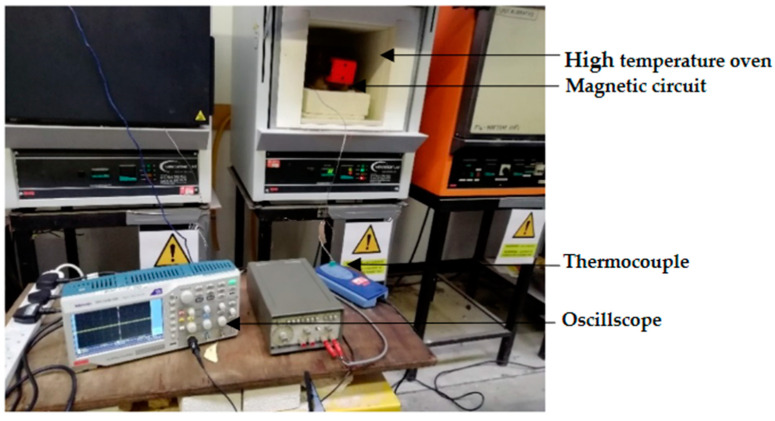
Measurement setup for high temperature testing.

The measurement system depicted in Figure 5 consists of two distinct environments, namely high-temperature and room temperature. The magnetic circuit is placed in the high-temperature environment, and a DC power supply and oscilloscope are placed in the room temperature environment. The magnetic circuit consists of the following components: An AlNiCo magnet, a non-magnetic metallic holder and the MFL coil sensor, all housed within an oven. The DC power supply is used to supply voltage to the circuit and the oscilloscope is used to detect the voltage changes. Using Autodesk Inventor, a holder was designed and machined based on the configuration of the ferrite core. The sensor is placed and positioned in a non-magnetic metallic holder made of Aluminium grade 91, which can withstand higher temperatures. A thermocouple is attached to the magnetic circuit inside the oven to test temperature stability. Picologger software is used to monitor thermocouple readings at room temperature. Figure 6 shows the positioning of the components in the experimental setup.

All of the components inside the oven, except for the ferrite core, have a maximum operating temperature of over 200 °C. The efficient functioning of the magnetic circuit at the stated condition is allowed by two separate environment measurement setups.

An initial test was carried out with by increasing the temperature from 32 °C with a progressive phase of 10 °C. The difference in voltage displayed inside the electronic equipment was noted. The measurements were taken at temperatures up to 200 °C, with the ferrite core working at 180 °C. It was noted that the output voltage obtained with the coil sensor for each varying thickness of the pipe sample decreased continuously with room temperature. Furthermore, the output voltage decreased with increasing temperature until 200 °C.

### 5.3. Coil Impedance

**Figure 7:**
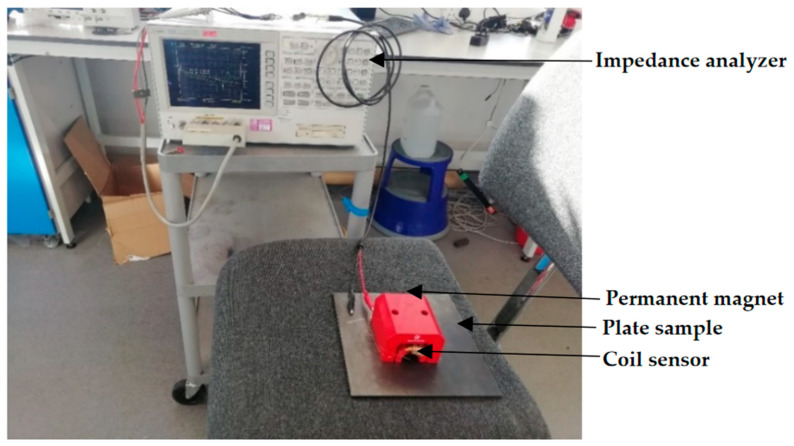
Coil sensor tested with impedance analyzer.

**Figure 8:**
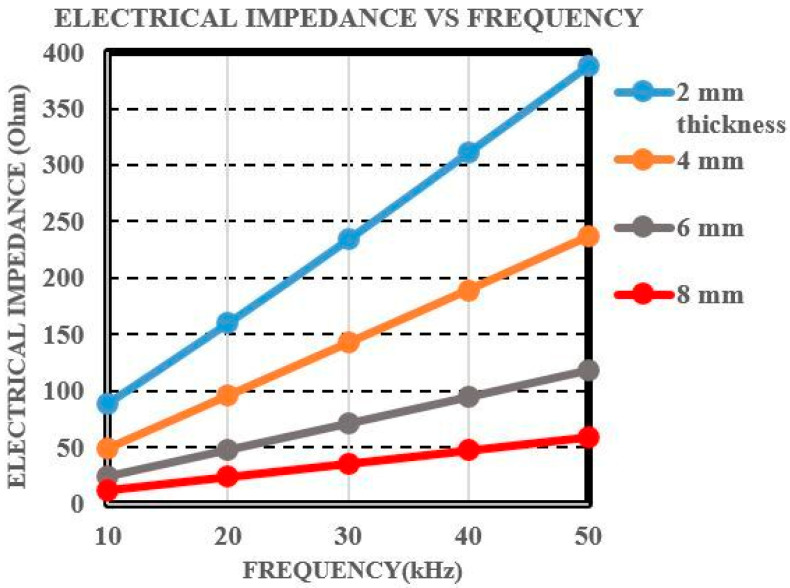
Electrical impedance plot for the varying plate sample thickness.

The coil impedance, as well as the number of wire loops wound around the ferrite core, are important parameters to consider in an MFL coil sensor. A basic test as shown in Figure 7 was carried out with an impedance analyzer, in which plate samples of thicknesses 2, 4, 6, and 8 mm were used with varying frequency while the positions of the magnet and coil sensor were kept fixed. Later pipe samples were considered for the experimental testing at room and high temperature conditions. Tests were carried out for up to a frequency of 50 kHz, and the electrical impedance at each frequency was determined for the 2–8 mm thick plate samples accordingly. The results obtained are shown in Figure 8. As noted the electrical impedance increases with frequency, and that at each frequency the impedance decreases with increasing the sample thickness. Since the measurements were conducted at low frequencies, the thickness of each plate sample was determined with less variation. As noted, electrical impedance values indicate that the MFL coil sensor can penetrate through plate sample thickness of 8 mm sample.

A sensing coil with less turns wound results in a much more compact coil with less occupied space, as well as reduced thermal heat. Furthermore, since copper wires have lower number of turns, printing them on a small circuit board is much easier. As a result, a coil sensor with 200 turns of wire cannot only be easily printed and fitted onto a small circuit board, but it can also detect small cross-sectional losses with ease, and therefore a coil sensor with 200 turns of wire was considered [43].

### 5.4. Magnitude of Magnetic Flux Density

**Figure 9:**
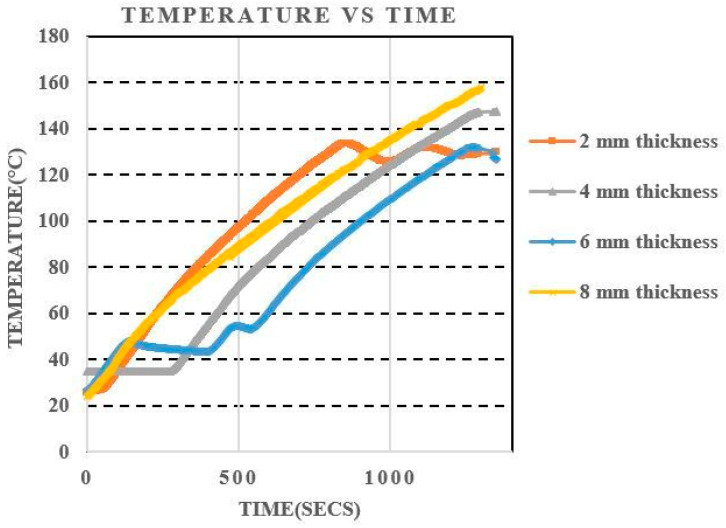
Temperature vibration over time for pipe samples of different thickness.

Using Picologger software, the temperature of the thermocouple connected to the pipe sample inside the oven was recorded at each second over time. The temperature increased in lockstep with the passage of time. The results obtained are shown in Figure 9.

**Figure 10:**
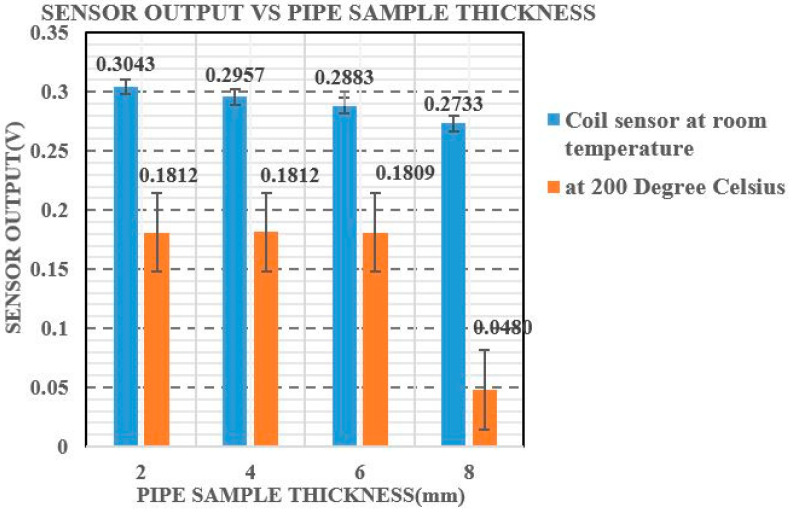
Comparison of the output voltage of the MFL coil sensor at room temperature and at 200 °C.

Further tests were carried out on the pipe samples at room temperature and at 200 °C, and the results are shown in Figure 10.

The relationship between changes in voltage and varying pipe sample thickness is found to be linear. Magnetic sensors transform magnetic induction signals obtained into voltage signals, which are observed using an oscilloscope [43].

As noted in Figure 10, the output voltage varies for each thickness of pipe sample at both temperature conditions. The difference in output voltage between 2 and 4 mm thick pipe samples was found to be 0.003563 V at room temperature, and between 4 mm and 6 mm thick samples was found to be 0.006627 V. The change in voltage observed between 6 and 8 mm thick pipe samples was 0.083568 V. As compared to other pipe sample thicknesses, the voltage difference between room temperature and 200 °C for 8 mm thick sample was vastly different. Since the ferrite core’s highest operating temperature is 180 °C, there is a greater difference in output voltage at high temperatures for 8 mm pipe sample thickness compared to room temperature.

## 6. Conclusions

The aim of this research was to create a magnetic circuit with a coil sensor mounted on pipe samples of different thicknesses that could be used to detect metal loss using the Magnetic Flux Leakage testing. A 3D FE model of the magnetic circuit has been developed taking into account the MFL coil sensor and sample geometry. A new MFL coil sensor for detecting thickness variation in pipes has been designed, developed and experimentally evaluated. The measurement set up is found to be suitable for increasing temperature conditions, with a suitable holder for the magnetic sensor’s positioning. The proposed method effectively tests metallic samples up to 8 mm for increasing temperature conditions. It has also been shown that the influence of temperature on the coil sensor does not cause any changes in voltage within the temperature range measured, and that the noise factor plays a significant role in voltage variation.

The 3D FE model can be further developed for high temperature conditions by including an additional physics module. Further research will be carried out investigations with this newly developed MFL coil sensor on pipe samples of higher thickness and at temperatures of up to 200 °C.

## Figures and Tables

**Table 1:** Specifications of the ferrite core [40].

Characteristics	Values
Ferrite core	T175-2
Reference permeability(µ_0_)	10
Material Density	5.0 g/cm^3^
Temperature coefficient of permeability(+ ppm/°C)	95
Co-efficient of linear expansion (+ ppm/°C)	10

## Data Availability

Data available on request due to restrictions eg privacy or ethical.
